# Pd^0^-Catalyzed Methyl Transfer on Nucleosides and Oligonucleotides, Envisaged as a PET Tracer

**DOI:** 10.3390/molecules181113654

**Published:** 2013-11-05

**Authors:** Damien James, Jean-Marc Escudier, Magali Szlosek-Pinaud, Eric Fouquet

**Affiliations:** 1 Institut des Sciences Moléculaires, Université de Bordeaux, UMR5255, 351 Cours de la Libération, Talence 33405, France; E-Mail: d.james@u-bordeaux1.fr; 2 Laboratoire de Synthèse et Physicochimie- de Molécules d’Intérêt Biologique, Université Paul Sabatier, UMR5068, 118 route de Narbonne, Toulouse 31062, France; E-Mail: escudier@chimie.ups-tlse.fr

**Keywords:** Stille coupling, nucleosides, oligonucleotides, methyl transfer, Positron Emission Tomography

## Abstract

The methyl transfer reaction from activated monomethyltin, via a modified Stille coupling reaction, was studied under “ligandless” conditions on fully deprotected 5'-modified nucleosides and one dinucleotide. The reaction was optimized to proceed in a few minutes and quantitative yield, even under dilute conditions, thus affording a rapid and efficient new method for oligonucleotide labelling with carbon-11.

## 1. Introduction

Coupling reactions mediated by transition metal catalysis are particularly powerful methods for the creation of carbon-carbon bonds. These reactions are especially recognized for combination of the mildness of the reaction conditions and their high efficiency and selectivity. Among them, palladium is undoubtedly the metal of choice and has been extensively used in the past decades in applications ranging from organic methodology development to total synthesis of complex structures. In recent years the field of applications has extended to the functionalization of biomolecules, mainly due to the ability of these coupling reactions to procede without any protection/deprotection sequences. In that context Positron Emission Tomography (PET) represents a field of choice for the application of the original palladium-catalysed coupling reactions. Indeed, PET is a powerful imaging technique for clinical, medical and biological investigations in various areas such as oncology, cardiology, and neurosciences, as well as for drug development. Due to the increasing need of this technique in *in vivo* biochemistry and medicine, the development of new tracers and radiolabelling strategies is always in demand [1,2,3], as outlined in recent reviews for the two most commonly used short half-life radioisotopes, e.g., carbon-11 (*t_1/2_* = 20.4 min) [4] and fluorine-18 (*t_1/2_* = 109.6 min) [5,6]. As a consequence, simple and rapid synthetic processes including organic transformations and purifications are required. Importantly, such a strategy becomes even more challenging when the biomolecule used as substrate is available in very low quantity, as the coupling reaction has to meet four mandatory constraints: (i) the selectivity of the reaction which is imposed by the presence of several functionalities and the absence of protecting groups, (ii) the mildness of the conditions required by the fragility of most of the substrates, (iii) the rapidity of the reaction which is directly related to the short half-live of the isotopes, especially for carbon-11, and finally (iv) the efficiency of the reaction which may have to occur under extremely dilute conditions either of the radiotracer and/or the biomolecule to be labeled.

In recent years, an increase of the use of palladium-mediated reactions for labeling purposes was observed [7]. Among these reactions, the palladium-mediated Stille reaction has proved to be an important route for the synthesis of PET radiotracers [8,9,10]. At this point, it is essential to note that most of the restrictions encountered in pharmaceutical industry, when using organotins for the synthesis of bioactive molecules, are not a problem anymore when applied to radiochemistry. Thus the palladium-catalysed Stille coupling still remains a topical reaction for the synthesis of radiotracers. It usually involves the reaction of [^11^C]-methyliodide with an aryl triorganostannane leading to a ^11^C-carbon-carbon coupled product. However, the preparation of an organostannyl precursor is not always straightforward, and in the case of functionalized organostannanes, nucleophilic groups have to be protected in order to prevent methylation as a side reaction [11]. Finally, difficulties might be encountered in separating the tracer from triorganotin residues.

**Scheme 1:**
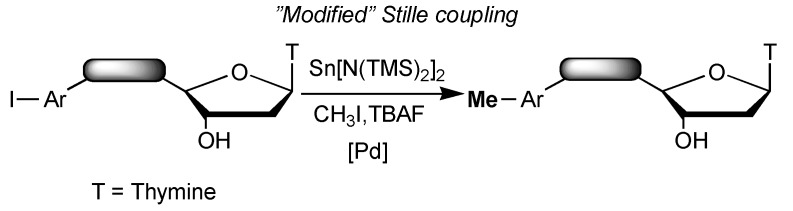
Methyl transfer reaction.

Recently, we have described a new ^11^C-labeling methodology based on the transfer reaction of the [^11^C]methyl group, from the ^11^C-labeled hypervalent methylstannate, first onto simple aryl halides [12] and then onto polyfunctional and heteroaromatic tracers for central nervous system [13]. Although the preparation of the labeled methyltin reagent is made from [^11^C]-methyl iodide, this new approach offers several advantages such as the ligand-free conditions and the formation of a nontoxic and easily removable inorganic tin by-product [14]. Our next interest was to investigate the applicability of this methodology for the labelling of important biomolecules such as nucleosides and oligonucleotides [15] which present an additional challenge in terms of available quantities (which are often tiny due to the costly and time consuming process for their synthesis), limiting until now the usefulness and expansion of such radiotracer. As a first approach, the study of the feasibility of the cross-coupling reaction under rapid and dilute conditions, on unprotected nucleosidic and dinucleotidic substrates bearing an iodoaryl moiety at the 5' position, with unlabelled methyl iodide is described herein (Scheme 1).

## 2. Results and Discussion

**Figure 1:**
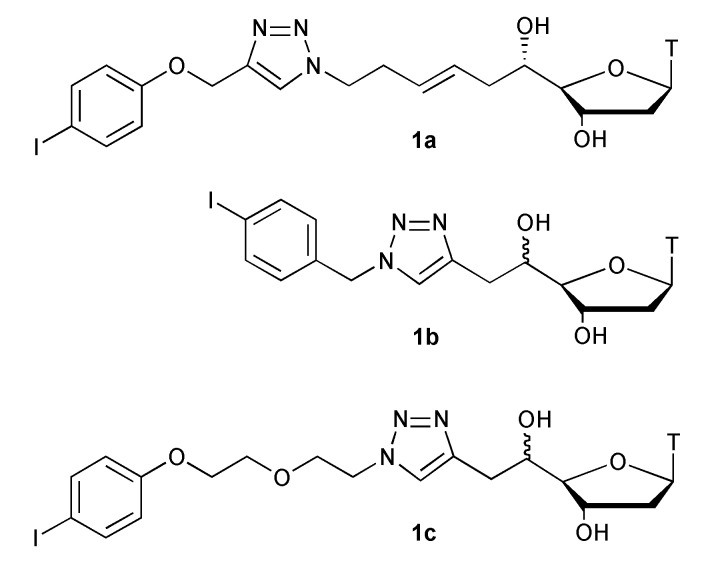
Substrates for the methyl transfer study.

Starting from thymidine aldehyde, we have synthesized 5'C-substituted thymidine derivatives either with alkyne or azide groups and then engaged them into a conjugation reaction with iodoaryl moieties by “click chemistry’’ leading to compounds **1a**–**c** (Figure 1) [16]. They were then used as substrates for the methyl transfer study. The monomethyltin reagent was prepared from iodomethane and Lappert’s stannylene [17,18] (Sn[N(TMS)_2_]_2_) and activated *in situ* with TBAF (tetrabutylammonium fluoride) giving the corresponding methylstannate, according to the previously described procedure [12].

Previous results led us run the reaction at 100 °C, using tris-(dibenzylideneacetone)dipalladium as catalyst (Pd_2_dba_3_, 10 mol%) under so-called “ligand free” conditions. Furthermore, due to the solubility properties of the substrates, the palladium-catalysed cross-coupling reaction was studied first in DMF instead of dioxane. In order to minimize reaction times and optimize the reaction yields, the concentration of the substrate was varied and the addition of CuI was studied (Table 1). Initially, the reaction was done using **1a** (0.2 M) as substrate without CuI (entry 1). In this case, a total conversion was observed in 5 min leading to the desired product **2a**, along with some degradation estimated to be about 20% and the formation of the corresponding hydrogenated by-product **3a**, resulting of the dehalogenation reaction of **1a**, in a 72/28 ratio in favour of **2a**. Interestingly, the addition of 20 mol% of CuI inhibited the degradation, but diminished the rate of the reaction, so that a total conversion was only attained in 50 min, and the formation of **3a** was still observed in a 63/37 ratio in favour of **2a** (entry 2). Under the same conditions, the increase of the concentration of **1a** (0.5 M) allowed us to get total conversion in 20 min and dramatically diminished the formation of **3a** (entry 3). Finally the use of 40 mol% of CuI impressively decreased the reaction time to only 5 min, with a good **2a**/**3a** ratio (92/8, entry 4). Those optimized conditions were then applied to **1b**, allowing also a total conversion in 5 min, but still with some amount of the corresponding hydrogenated product **3b** (entry 5).

**Table 1:** Methyl transfer reactions onto **1a**‒**c**. 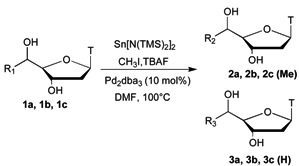

Entry	Compd.[mol.L^−1^]	CuI [mol%]	Time ^[a]^ [min]	2/3 ^[b]^
1	**1a** (0.2)	0	5	**2a**/**3a**: 72/28^ [c]^
2	**1a** (0.2)	20	50	**2a**/**3a**: 63/37
3	**1a** (0.5)	20	20	**2a**/**3a**: 97/3
4	**1a** (0.5)	40	5	**2a**/**3a**: 92/8
5	**1b** (0.5)	40	5	**2b**/**3b**: 80/20
6	**1c** (0.5)	40	50	**2c**/**3c**: 100/0
7	**1c** (0.5)	140	5	**2c**/**3c**: 100/0

^[a]^ For total conversion. ^[b]^ Estimated by HPLC. ^[c]^ With degradation estimated to 20%.

Finally, when the optimized conditions of **1a** were applied to **1c**, a total conversion was observed after 50 min, but in this case, the sole methylated compound **2c** was formed (entry 6). In order to explain the increase of the reaction time, we could envisage that the copper is coordinated by the oxygen atoms of the ethylene glycol arm keeping it unavailable for the catalytic cycle of the Stille coupling. Indeed, the use of 140 mol% of CuI allowed us to recover a total conversion in 5 min again with the sole formation of the desired compound **2c** (entry 7). Interestingly, the use of only 5 mol% of Pd_2_dba_3_ with 20 mol% of CuI led to total conversion in 5 min with a better **2b**/**3b** ratio of 93/7 under classical heating conditions (to be compared to entry 5), and in 2 min with a **2b**/**3b** ratio of 96/4 under microwave activation.

Thus, having in hand conditions allowing a few minutes reaction time, compatible with the half-life of carbon-11 and unprotected nucleosides, we wanted to extend this methodology to oligonucleotides (ODNs). The first difficulty was related to the concentration of the substrate and the palladium charge. Indeed, ODNs are produced in such small quantities that running a methyl transfer reaction using an ODN concentration of 0.5 M and 10 mol% of Pd_2_dba_3_ was not possible. The second point was the low solubility of ODNs in DMF. Finally, the third question concerned the compatibility of our conditions with the phosphodiester linkage of ODNs. In order to address those three questions, we studied first the methyl tranfer on **1b** at 5 × 10^−3^ M, with 100 mol% of Pd_2_dba_3_, at 100 °C in various solvents under classical or microwave heating conditions (Table 2). The direct transposition of the previous conditions using a Pd/Cu ratio of 1/2 (entry 1) led to a strong decrease of the reaction rate, as the total conversion was only observed after 60 min with a similar ratio **2b**/**3b**. Furthermore, the increase of the amount of CuI totally inhibited the reaction (entry 2). On the contrary, the absence of CuI allowed to recover a total conversion in 5min with a better **2b**/**3b** ratio of 90/10 (entry 3). Under the same conditions, the use of microwave irradiation instead of classical heating did not result in any improvements (entry 4).

**Table 2:** Methyl transfer onto **1b** under dilute conditions (5 × 10^−3^ M).

Entry	Solvent	CuI [mol%]	MW	Time [min] ^[a]^	2/3 ^[b]^
1	DMF	400	no	60	81/19 ^[c]^
2	DMF	2000	no	-	-
3	DMF	0	no	5	90/10
4	DMF	0	yes	5	89/11
5	DMF/DMSO 9/1	0	no	5	66/34 ^[c]^
6	DMF/DMSO 9/1	0	yes	5	96/4
7	DMF/DMSO 3/1	0	yes	5	91/9
8	DMF/H_2_O 9.5/0.5	0	no	40	54/46 ^[c]^
9	DMF/H_2_O 9.5/0.5	0	yes	5	85/15
10	DMF/H_2_O 9/1	0	yes	5	60/40

^[a]^ For total conversion. ^[b]^ Estimated by HPLC. ^[c]^ With impurities estimated to less than 10%.

However, addition of 10% DMSO in DMF, under classical heating led to an increased formation of the hydrogenated product **3b** (34%) together with apparition of side products (entry 5), while microwave heating, under the same conditions (entry 6), allowed us to get a clean reaction with a 96/4 **2b**/**3b** ratio. The increase of the proportion of DMSO under microwaves (entry 7), led to an almost similar **2b**/**3b** ratio. The use of H_2_O instead of DMSO, even in a very small quantity, under classical heating, seriously disturbed the rate of the reaction and the formation of the methylated compound (entry 8). The use of microwaves in the same conditions allowed us to recover a total conversion in 5 min with quite a good **2b**/**3b** ratio (entry 9). Finally, even a slight increase of the proportion of H_2_O was not appropriate at all (entry 10). After the achievement of the procedure optimization on the monomeric substrates, the last step was to test our best conditions on the dinucleotide **4** synthesized as a 70/30 mixture of diastereomers according to the standard phosphoramidite method from the 5'C-substituted nucleoside from **1b** [19,20].

**Scheme 2:**
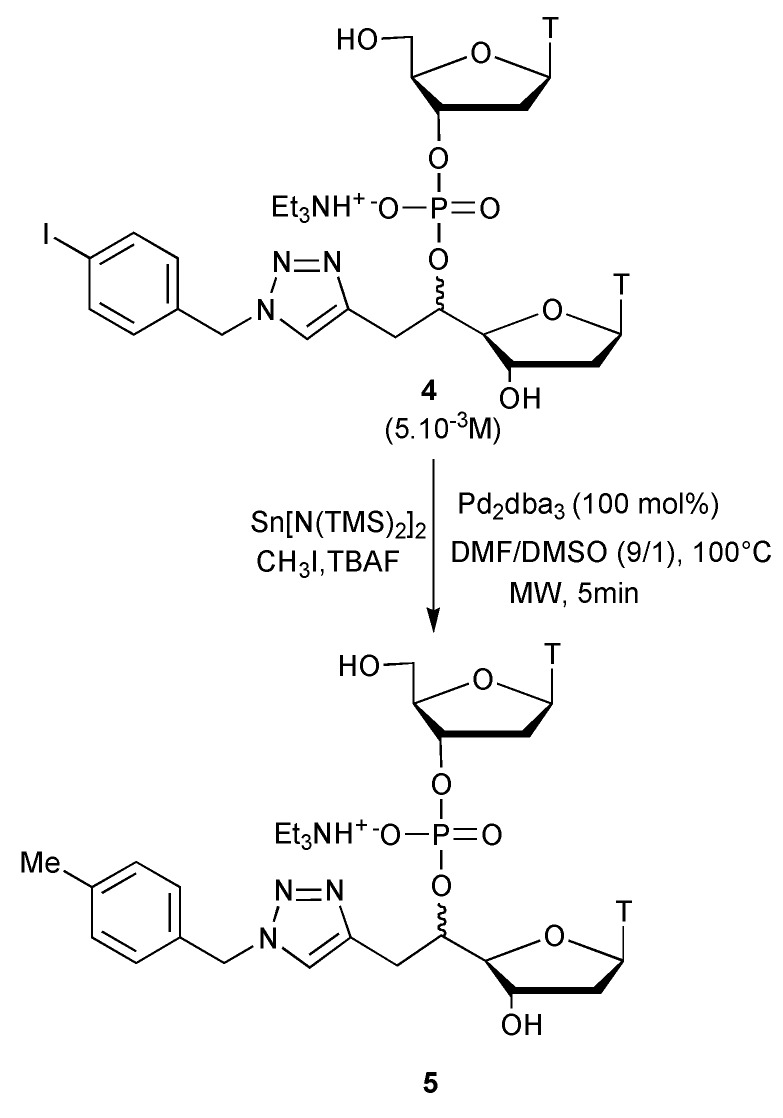
Methyl transfer study onto dinucleotide **4**.

Thus, **4** was used at a 5 × 10^−3^ M concentration in a mixture of DMF/DMSO (9/1), with 100 mol% of Pd_2_dba_3_ (Scheme 2). The reaction was run at 100 °C, under microwave heating, leading to a total conversion into the single desired methylated compound **5** in 5 min. Noteworthily, the increase of the DMSO/DMF ratio up to 1/1 only slightly perturbed the reaction, as the total conversion was still obtained in 5 min, but we could observe the formation of the corresponding hydrogenated compound in a 87/13 ratio in favour of **5**.

## 3. Experimental

### 3.1. General

All water-sensitive reactions were carried out under a nitrogen atmosphere with dry solvents under anhydrous conditions. Yields refer to chromatographically and spectroscopically (^1^H NMR) homogeneous materials. Macherey Nagel silica gel 60M (230–400 mesh ASTM) was used for flash chromatography. CH_2_Cl_2_ and (*i*-Pr)_2_NH were distilled over CaH_2_. THF and Et_2_O were distilled from sodium and benzophenone. Ethanol and methanol were dried over magnesium turnings activated by iodine. Micro-wave assisted reaction were carried out on a Biotage Initiator. HPLC was performed on a Waters 600 system equipped with a Waters 996 photodiode array detector. Analytical and semi-preparative HPLC were performed with a reversed-phase column (Phenomenex Luna C18, 5 µm, 250 × 4.6 mm and Kromasil C18, 5 µm, 250 × 20 mm) using the following solvent systems at 1 mL/min and 4 mL/min: Acetonitrile (solvent A) and H_2_O MilliQ (solvent C) for reactions on modified thymidines, Acetonitrile (solvent A) and TEAA_aq_ (triethylammonium acetate) 50 mM at pH = 7 (solvent B) for reactions on modified dinucleotide. ^1^H-NMR and ^13^C-NMR were recorded on Bruker DPX-200 FT (^1^H: 200 MHz, ^13^C: 50.2 MHz), Bruker AC-250 FT (^1^H: 250 MHz, ^13^C: 62.9 MHz), Bruker AVANCE-300 FT (^1^H: 300 MHz, ^13^C: 75.5 MHz) and Bruker DPX-400 FT (^1^H: 400 MHz, ^13^C: 100.2 MHz) apparatus using indicated internal reference. The chemical shifts (δ) and coupling constants (*J*) are expressed in ppm and Hz respectively. Mass spectra were recorded on a Nermag R10-10C. High resolution mass spectra were performed by the CESAMO (Talence, France) and were recorded on a FT-IRC mass spectrometer Bruker 4.7T BioApex II. MALDI-MS spectra were performed by the CESAMO (Talence, France) on a Voyager mass spectrometer (Applied Biosystems).

### 3.2. Preparation of **4**

Dinucleotide **4** was prepared in four steps from the 3'-*O*-TBDPS derivative of **1b**, as follows:

#### 3.2.1. Coupling (**4DCT**)

The 3'-*O*-TBDPS derivative of **1b** (1.24 g, 1.6 mmol), the commercially available 3'-*O*-(2-cyanoethyl diisopropylphosphoramidite)-5'-O-DMTr-thymidine (4 g, 5.33 mmol, 3.3 eq.) and a solution of tetrazole (0.45 M in CH_3_CN, 47 mL, 210 mmol, 13 eq.) were stirred 30 min at room temperature. Then collidine (2.75 mL, 20.8 mmol, 13 equiv.) and an excess of a solution of I_2_ [0.1 M in THF/H2O (2:1)] were added. Then a solution of Na_2_S_2_O_3_ was added until the reaction mixture remained colorless. The mixture was diluted with 200 mL of ethyl acetate, washed with H_2_O (2 × 100 mL) and brine (100 mL). The organic layer was dried over MgSO_4_ and evaporated to dryness under vacuum. The crude product was purified by flash chromatography by flash purification (ethyl acetate/CH_2_Cl_2_ : 1/1 → 8/2 → 1/0) affording the all protected dinucleotide **4DCT** (1.78 g, 78% yield) as a white foam. ^1^H-NMR (400 MHz, CDCl_3_), δ_ppm_: 1.03 (s, 9H, CH_3_ *t*Bu), 1.35–1.39 (m, 3H, CH_3_ thymine), 1.82–1.93 (m, 4H, H_2'_ and CH_3_ thymine), 2.31–2.42 (m, 5H, H_2'_, H_2″_ and CH_2_CN), 2.51–2.54 (m, 1H, CH_2_CN CEO), 2.72–2.98 (m, 2H, H_6'_), 3.28–3.49 (m, 2H, CH_2_-O), 3.76–3.89 (m, 7H, H_5″_ and CH_3_ DMTr), 3.93–4.06 (m, 3H, H_5″_, H_4'_ and H_4″_), 4.21–4.32 (m, 1H, H_3″_), 4.40 (s, 1H, H_5'_), 5.01–5.04 (m, 1H, H_3'_), 5.41 (s, 2H, H_9'_), 6.23–6.30 (m, 1H, H_1″_), 6.45–6.55 (m, 1H, H_1'_), 6.83 (d, 4H, *J =* 5.9 Hz, CH DMTr), 6.98 and 7.03 (2d, 2H, *J =* 10.0 Hz, H_11'_ and H_15'_), 7.21–7.36 (m, 16H, H8', CH phenyl and CH DMTr), 7.53–7.69 (m, 8H, H_12'_, H_14'_, CH thymine and CH phenyl), 8.81 (s, 2H, NH thymine). ^13^C-NMR (100.2 MHz, CDCl_3_), δ_ppm_: 11.8 and 12.8 (CH_3_ thymine), 19.3 (C *t*Bu), 19.53 and 19.81 (CH_2_CN), 27.1 (CH_3_ *t*Bu), 29.0 (C_6'_), 38.9 and 39.0 (C_2″_), 40.1 and 40.1 (C_2'_), 53.6 and 53.7 (C_9'_), 55.5 (CH_3_ DMTr), 62.4 and 62.7 (C_5″_), 63.4 and 63.6 (CH_2_O), 74.6 (C_5'_), 79.2 and 80.1 (C_3'_ and C_3″_), 84.2 and 85.2 (C_1'_, C_1″_ and C_4″_), 87.5 (C_4'_), 94.7 and 94.8 (C_13'_), 111.6 and 112.2 (C thymine), 113.6 (CH DMTr), 116.4 and 116.9 (CN), 122.3 and 123.0 (CH DMTr), 127.5 (CH phenyl), 128.3 and 128.4 (CH DMTr), 129.4 (C_8'_), 130.1 and 130.2 (C_11'_ and C_15'_), 130.4 (CH phenyl), 132.7 and 133.1 (C phenyl and C_10'_), 134.6 and 135.5 (C DMTr), 135.9 (CH thymine and CH phenyl), 138.4 and 138.5 (C_12'_ and C_14'_), 142.6 (C DMTr), 144.2 (C_7'_), 150.8 and 151.1 (C DMTr and C=O thymine), 159.1 (C-OMe DMTr), 164.1 (C=O thymine). ^31^P-NMR (121.6 MHz, CDCl_3_), δ_ppm_: −3.63 and −3.06. MALDI-TOF, *m/z*: 1459.3 ([M+Na^+^]^+^), 1475.2 ([M+K^+^]^+^).

#### 3.2.2. Cleavage of DMTr Group (**4CT**)

To a solution of **4DCT** (450 mg, 0.31 mmol.) in CH_2_Cl_2_ (3 mL) was added TFA (90 µL, 1.2 mmol, 3.9 equiv.). The clearly red solution was stirred 30 min at room temperature. The solvent was evaporated under vacuum and the crude product was purified by flash chromatography (ethyl acetate/methanol: 100/0 → 95/5) affording **4CT** (350 mg, 98% yield) as a white foam. ^1^H-NMR (400 MHz, CDCl_3_), δ_ppm_: 1.03 (s, 9H, CH_3_ *t*Bu), 1.24 (m, 1H, H_6'_), 1.83 (s, 3H, CH_3_ thymine), 2.27–2.75 (m, 7H, H_6'_, H_2'_, H_2″_ and CH_2_CN), 3.74–4.01 (m, 7H, H_5'_, H_5″_,H_4'_, OH and CH_2_O), 4.40 (s, 2H, H_4″_ and H_3″_), 4.97 (m, 1H, H_3'_), 5.42 (s, 2H, H_9'_), 6.01–6.39 (m, 2H, H_1'_ and H_1″_), 7.01 (2d, 2H, *J =* 8.0 Hz, H_11'_ and H_15'_), 7.35–7.42 (m, 9H, H_8'_ and CH phenyl), 7.58–7.62 (m, 4H, CH phenyl and CH thymine), 7.68 (2d, 2H, *J =* 8.0 Hz, H_12'_ and H_14'_), 9.73 (s, 2H, NH thymine). ^13^C-NMR (100.2 MHz, CDCl_3_), δ_ppm_: 12.4 (CH_3_ thymine), 19.00 (CH_2_CN and C *t*Bu), 26.9 (CH_3_ *t*Bu), 29.8 (C_6'_), 38.3 (C_2'_ and C_2″_), 53.4 (C_9'_), 60.5 (C_5'_ and C_5″_), 62.7 (CH_2_O), 74.5 (C_4″_), 79.4 and 79.6 (C_3'_), 85.7 (C_1'_ and C_1″_), 87.7 (C_4'_), 94.8 (C_13'_), 111.8 (CN), 115.1 and 117.9 (C thymine), 123.3 (C_8'_), 128.3 (CH phenyl), 130.1 and 130.3 (C_11'_ and C_15'_), 132.8–133.1 (C phenyl), 134.6–134.7 (C_10'_), 135.9 (CH thymine), 138.4 (C_12'_ and C_14'_), 142.6 (C_7'_), 151.0 (C=O thymine), 164.5–164.7 (C=O thymine). ^31^P-NMR (121.6 MHz, CDCl_3_), δ_ppm_: −3.26. MALDI-TOF, *m/z*: 1157.4 ([M+Na^+^]^+^).

#### 3.2.3. Cleavage of Cyanoethyl Group (**4T**)

To a solution of **4CT** (190 mg, 0.17 mmol) in CH_3_CN (3.4 mL) was added Et_3_N (350 µL, 2.49 mmol, 15 equiv.). The reaction mixture was stirred 1h at 60 °C. The solvents were evaporated under vacuum affording **4T** under the triethylammonium salt form (170 mg, 86% yield) as a white foam. ^1^H-NMR (400 MHz, MeOD), δ_ppm_: 1.01 (CH_3_ *t*Bu), 1.28 (t, 9H, *J =* 8.0 Hz, CH_3_ Et_3_NH^+^), 1.88 and 1.89 (2s, 6H, CH_3_ thymidine), 2.07–2.23 (m, 4H, H_2'_ and H_2″_), 3.05 (t, 1H, *J =* 8.0 Hz, H_6'_), 3.17 (q, 7H, *J =* 8.0 Hz , H_6'_ and CH_2_ Et_3_NH^+^), 3.70 (s, 2H, H_5'_), 3.85 (s, 2H, H_4'_ and H_4″_), 4.01–4.09 (m, 1H, H_5'_), 4.61 (s, 1H, H_3'_), 4.77 (s, 1H, H_3″_), 5.51 (s, 2H, H_9'_), 6.11 (t, 1H, *J =* 4.0 Hz, H_1″_), 6.58 (t, 1H, *J = 8.0 Hz*, H_1'_), 7.08 (2d, 2H, *J =* 12.0 Hz, H_11'_ and H_15'_), 7.24–7.42 (m, 8H, CH phenyl), 7.56 (d, 2H, *J =* 8.0 Hz, CH phenyl), 7.64 (d, 2H, *J =* 8 Hz, H_12'_ and H_14'_), 7.77 (2s, 2H, H_8'_ and CH thymine), 7.89 (s, 1H, CH thymine). ^13^C-NMR (100.2 MHz, MeOD), δ_ppm_: 9.35 (CH_3_ HNEt_3_^+^), 12.7 and 12.9 (CH_3_ thymine), 27.6 (CH_3_ tBu), 29.7 (C_6'_), 40.1 (C_2'_) 41.1 (C_2″_), 46.9 (CH_2_ Et_3_NH^+^), 54.4 (C_9'_), 62.6 (C_5″_), 76.5 (C_3″_), 76.6 (C_5'_), 77.3 (C_3'_), 86.0 (C_1'_ and C_1″_), 86.8 (C_4'_), 87.5 (C_4″_), 95.1 (C_13'_), 111.6 and 112.3 (C thymine), 124.9 (C_8'_), 129.2 (C_11'_, C_15'_ and CH phenyl), 131.3 (C_10'_ and C phenyl), 136.8 (CH phenyl), 137.1 (CH phenyl), 138.3 (CH thymine), 139.4 (C_12'_ and C_14'_), 144.7 (C_7'_), 152.3 and 152.7 (C=O thymine), 166.4 (C=O thymine). ^31^P-NMR (121.6 MHz, MeOD) δ_ppm_: −2.14. MALDI-TOF, *m/z*: 1078.8 ([M–Et_3_NH^+^]^−^).

#### 3.2.4. Cleavage of TBDPS p: Preparation of Compound **4**

To a solution of **4T** (170 mg, 0.14 mmol, 1 eq.) in CH_3_CN (2 mL, HPLC grade) was added a solution of TBAF [(1 M in THF), 170 µL, 0.17 mmol, 1.2 equiv.]. The reaction mixture was stirred overnight at room temperature. The solvent was evaporated under vacuum and the crude product was purified by precipitation in Et_2_O affording **4**, under the triethylammonium salt form, **(**105 mg, 77% yield) as white foam. ^1^H-NMR (300 MHz, MeOD), δ_ppm_: 1.30 (t, 9H, *J =* 7.1 Hz, CH_3_ Et_3_NH^+^), 1.59–1.72 (m, 2H, H_6'_), 1.86 (s, 3H, CH_3_ thymine), 1.93 (s, 3H, CH_3_ thymine), 2.20–2.51 (m, 4H, H_2'_ and H_2″_), 3.15–3.27 (m, 7H, H_5'_ and CH_2_ Et_3_NH^+^), 3.79–3.83 (m, 3H, H_4'_ and H_5″_), 4.11–4.16 (m, 1H, _4″_), 4.57–4.72 (m, 2H, H_3'_ and H_3″_), 5.54 (s, 2H, H_9'_), 6.19–6.43 (m, 2H, H_1'_ and H_1″_), 7.07 (d, 2H, *J =* 8.3 Hz, H_11'_ and H_15'_), 7.67 (d, 2H, *J =* 7.9 Hz, H_12'_ and H_14'_), 7.85 (d, 1H, *J =* 5.6 Hz, CH thymine), 7.93–7.96 (m, 2H, H_8’_ and CH thymine). ^13^C-NMR (75.5 MHz, MeOD), δ_ppm_: 9.2 (CH_3_ Et_3_NH^+^), 12.6 and 12.9 (CH_3_ thymine), 24.7 (C_6'_), 40.0 and 40.6 (C_2'_ and C_2'_), 47.6 (CH_2_ Et_3_NH^+^), 54.1 (C_9'_), 59.4 (C_5'_), 62.8 (C_5″_), 72.1 and 77.1 (C_3'_ and C_3″_), 85.5 and 86.0 (C_1'_ and C_1″_), 87.6 and 88.0 (C_4'_ and C_4″_), 94.8 (C_13'_), 111.5 and 112.1 (C thymine), 125.7 (C_8'_), 131.1 (C_11'_ and C_15'_), 136.7 and 138 (C_7'_, C_10'_ and CH thymine), 139.1 (C_12'_ and C_14'_), 152.17 and 152.3 (C=O thymine), 166.2 (C=O thymine). ^31^P-NMR (81.2 MHz, MeOD), δ_ppm_: −0.63. MALDI-TOF, *m/z*: 843.9 ([M+2H^+^–Et_3_NH^+^]^+^), 865.9 ([M+H^+^ + Na^+^–Et_3_NH^+^]^+^). HPLC: rt = 17.15 and 17.81 min (A/B: from 20/80 to 30/70 in 15 min then to 50/50 in 2 min then to 50/50 during 2 min then to 20/80 in 1 min).

### 3.3. General Procedure for Methylation (0.2 and 0.5 M)

According to the previously described procedure [12] the monomethylstannane was prepared from Lappert’s stannylene and iodomethane. To a solution of monomethylstannane (2 equiv.) in THF (0.5 M), was added a commercial solution of TBAF 1M in THF (6 equiv.). The colourless resulting solution was stirred for 5 min and the solvent was removed under reduced pressure. The residue was dissolved in DMF (0.2 or 0.5 M). Pd_2_dba_3_ (10 mol%), CuI (0–140 mol%) and the electrophile (**1a**, **1b** or **1c**, 1 equiv.) were added. The reaction mixture was stirred at 100 °C until total consumption of the starting material followed by HPLC (Phenomenex Luna C18, 5 mm, 250 × 4.6 mm). After cooling, the reaction mixture was diluted with methanol and the precipitate was filtrated. Solvents were evaporated under vacuum and the crude product was purified by semi-preparative HPLC (Kromasil C18, 5 mm, 250 × 20 mm).

*(5'S)-C-(1-(Pent-3-enyl)-(4-(4-methylphenoxy)-methyl)-1H-1,2,3-triazol-1-yl)-thymidine* (**2a**). HRMS (ESI), *m/z* calculated for C_25_H_31_N_5_O_6_Na ([M+Na^+^]^+^) 520.2166, found 520.2174. HPLC: rt = 9.94 (A/C: from 30/70 to 70/30 in 15 min then to 90/10 in 1 min then to 40/60 in 1 min).

*5'-C-(1-(4-Methyl-benzyl)-4-methyl-1H-1,2,3-triazol-4-yl)-thymidine* (**2b**). ^1^H-NMR (300 MHz, MeOD), δ_ppm_: 1.85 (s, 3H, CH_3_ thymine), 2.19–2.22 (m, 2H, H_2'_), 2.29 (s, 3H, CH_3_), 2.81–3.04 (m, 2H, H_6'_), 3.76–3.81 (m, 1H, H_4'_), 4.00–4.10 (m, 1H, H_5'_), 4.40–4.56 (m, 1H, H_3'_), 5.48 (s, 2H, H_9'_), 6.22–6.32 (m, 1H, H_1'_), 7.13–7.21 (m, 4H, H_11'_, H_12'_, H_14'_ and H_15'_), 7.67 (s, 0.3H, CH thymine), 7.75–7.76 (m, 1H, H_8'_), 7.96 (s, 0.7H, CH thymine). ^13^C-NMR (75.5 MHz, MeOD), δ_ppm_: 12.5 (CH_3_ thymine), 21.2 (CH_3_), 31.5 (C_6'_), 41.0 (C_2'_), 54.7 (C_9'_), 71.5 (C_5'_), 73.2 (C_3'_), 86.2 (C_1'_), 89.6 and 90.5 (C_4'_), 111.6 (C thymine), 124.4 (C_8'_), 129.1 (C_11'_ and C_15'_), 130.6 (C_12'_ and C_14'_), 133.8 (C_13'_), 138.5 (CH thymine), 139.5 (C_10'_), 146.0 (C_7'_), 152.4 (C=O thymine), 166.4 (C=O thymine). HRMS (ESI) *m/z* calculated for C_21_H_26_N_5_O_5_ 428.1928, found 428.1934. HPLC: rt = 5.75 min (A/C: from 30/70 to 70/30 in 15 min then to 90/10 in 1 min then to 40/60 in 1 min).

*5'-C-(1-(2-(2-(4-Methylphenoxy)ethoxy)ethyl)-4-methyl-1H-1,2,3-triazol-4-yl)-thymidine* (**2c**). MS: 502.23 ([M+H^+^]^+^), 524.22 ([M+Na^+^]^+^). HPLC: rt = 8.09 min (A/C: from 40/60 to 70/30 in 10 min then to 90/10 in 3 min then to 40/60 in 1 min).

*(5'S)-C-(1-(Pent-3-enyl)-(4-phenoxy)-methyl)-1H-1,2,3-triazol-1-yl)-thymidine* (**3a**). MS (ESI): 483.24 ([M+H^+^]^+^), 506.20 ([M+Na^+^]^+^). HPLC: rt = 7.04 min (A/C: from 30/70 to 70/30 in 15 min then to 90/10 in 1 min then to 40/60 in 1 min).

*5'-C-(1-Benzyl-4-methyl-1H-1,2,3-triazol-4-yl)-thymidine* (**3b**). ^1^H-NMR (300 MHz, DMSO *d_6_*), δ_ppm_: 1.77 (s, 3H, CH_3_ thymine), 2.05–2.10 (m, 2H, H_2'_), 2.68–2.92 (m, 2H, H_6'_), 3.36 (s, 2H, OH), 3.66–3.69 (m, 1H, H_4'_), 3.82–3.95 (m, 1H, H_5'_), 4.28 (major 0.7) and 4.41 (minor 0.3) (2s, 1H, H_3'_), 5.19–5.30 (m, 2H, OH), 5.55 (s, 2H, H_9'_), 6.19–6.22 (m, 1H, H_1'_), 7.29–7.38 (m, 5H, H phenyl), 7.64 (s, 1H, H_8'_), 7.93 (s, 1H, CH thymine), 11.28 (s, 1H, NH thymine). ^13^C-NMR (75.5 MHz, DMSO *d_6_*), δ_ppm_: 12.4 (CH_3_ thymine), 30.3 (C_6'_), 40.6 (C_2'_), 52.6 (C_9'_), 69.7 (C_5'_), 71.3 (C_3'_) 83.7 (C_1'_), 87.9 (C_4'_), 89.0 (C_4'_), 109.3 (C thymine), 123.1 (C8'), 127.8 and 128.7 (CH phenyl), 136.3 (C phenyl and C thymine), 144.2 (C_7'_), 150.4 (C=O thymine), 163.7 (C=O thymine). HRMS (ESI) *m/z* calculated for C_20_H_24_N_5_O_5_ ([M+H^+^]^+^) 414.1771, found 414.1777. HPLC: rt = 4.01 min (A/C: from 30/70 to 70/30 in 15 min then to 90/10 in 1 min then to 40/60 in 1 min).

### 3.4. Methylation under Dilute Conditions (5 × 10^−3^ M)

According to the previously described procedure [12] the monomethylstannane was prepared from Lappert’s stannylene and iodomethane. To a solution of monomethylstannane (18.0 mg, 0.032 mmol, 2 equiv.) in THF (300 µL) was added a commercial 1 M solution of TBAF in THF (96 µL, 6 equiv.). The colourless solution was stirred for 5 min and the solvent was removed under reduced pressure. The residue was solubilized in 3.2 mL of a DMF/DMSO 9/1 mixture, then 15 mg of Pd_2_dba_3_ (100 mol%) and 15 mg of **4** (0.016 mmol, 1 equiv.) were added. The reaction mixture was stirred for 5 min at 100 °C under microwave conditions. The total conversion, was confirmed by analytical HPLC (Phenomenex Luna C18, 5 mm, 250 × 4.6mm). After cooling, the reaction mixture was diluted with methanol and the precipitate was filtrated. Solvents were evaporated under vacuum and the crude product **5** was purified as a mixture of diastereomers by semi-preparative HPLC (Kromasil C18, 5 mm, 250 × 20 mm). MALDI-TOF: 732.1 ([M–Et_3_NH^+^ + 2H^+^]^+^), 754.1 ([M–Et_3_NH^+^ + H^+^ + Na^+^]^+^). HPLC: rt = 12.52 and 12.98 min (A/B: from 20/80 to 30/70 in 15 min then to 50/50 in 2 min then to 50/50 during 2 min then to 20/80 in 2 min).

## 4. Conclusions

In summary, we have demonstrated that our palladium-catalysed methyl transfer reaction could be a very fast and efficient way for the direct carbon-11 labeling of unprotected nucleosidic and oligonucleotidic substrates, even under dilute conditions. Furthermore, we are currently investigating the use of [^11^C]-monomethylstannate, prepared according to our previous work [12] under the new coupling conditions herein developed for labelling **1b** and **4**, as well as for modified monomers **1a**, **1b** and **1c** incorporated into model oligonucleotide sequences [21,22].
